# Supplementary material for: Emotionally congruent music and text increase immersion and appraisal
Source: PLoS One. 2023 Jan 12;18(1):e0280019. doi: 10.1371/journal.pone.0280019 (PMC9836297; doi:10.1371/journal.pone.0280019)
Supplement: S1 Table — (DOCX) [file pone.0280019.s001.docx]

**S1 Table. List of novel excerpts presented as text stimuli derived from Zupan and Babbage [2].**

| Author and Title | G.H. Ephron: Guilt | Emily Giffin: Baby Proof | Christopher Coake: We’re in trouble | Jodi Picoult: My Sister’s Keeper |
| --- | --- | --- | --- | --- |
| Context | Luke has invited Abbey’s sister Annie and her boyfriend Peter to dinner, in hopes that he can surprise Abbey with a proposal of marriage. | Claudia is on her way to meet her ex-husband, Ben, at a restaurant for lunch. She hopes that they will be able to reconcile their differences because she is still in love with him. | Eric begins to tell Kristen, the love of his life, about his most vivid memory as a child. | Kate has not heard from her boyfriend, Taylor, who she met when they were both receiving Chemotherapy in the hospital. |
| Content | Abbey joins her boyfriend Luke at a restaurant for  dinner. She is surprised to find her sister and other friends also there and even more surprised when Luke proposes to her after only a few months of dating and excitedly accepts his proposal. | Claudia enters the restaurant prepared to congratulate Ben after assuming he has recently become engaged to a girl he was dating. She is surprised when he announces that he is not engaged because he still loves her. | Eric tells the story of playing with his dog, Gale, while in a park at seven years old. He plays catch with Gale, throwing the ball numerous times as Gale got increasingly more excited about the game. Eric throws the ball harder and the ball bounces off the edge of a cliff. Gale follows the ball over the edge and Eric makes it to the cliff’s edge just in time to see Gale fall. | Kate is telling her mother that she has not heard from  her boyfriend in a few days and she thinks he is mad at her. After inquiring at the nurse’s desk, Kate’s mother finds out that Taylor had died and she makes the painful decision to not tell her daughter until her chemotherapy has been completed. Kate is devastated when her mother finally shares the news one month later. |
| Excerpt | Chapter 13  - Par. 24 “The restaurant was just … Italian country villa.” - Par. 28 “Luke waved from … a white shirt.” - Par. 29 “Annie introduced him … the baby giraffe.” - Par. 40 “’It’s this big …doing here anyway?” - Par. 45 “Her voice died …Will you marry me?” - Par. 56 “Abby’s hands trembled … looked over at Luke.” - Par. 59 “’Yes! Yes! Yes! …on her finger.” | Chapter 31  - Par. 18 “Forty-five minutes later … out from my mind.” - Par. 19 “As I walk … sad all at once.” - Par. 21 “It suddenly occurs … ‘No work today.’” - Par. 34 “Ben fills the silence … of his engagement?” - Par. 36 “’Yeah’, he says … big of you.’” - Par. 38 “I manage a small … to be happy.’” - Par. 43 “I want you … so many times.” - Par. 48 “I look at him … says no, that” - Par. 53 “she’s engaged … guy named Steve” - Par. 57 “I feel a surge … dating Richard anymore.” - Par. 61 “Ben breaks into … renders me speechless.” - Par. 65 “We are quiet … do anything for you.’” | Chapter 1  - Par. 4 “Kristen says in … that is you.’” - Par. 8 “Tell me the most …you can remember.” - Par. 9 “Eric is quiet.” - Par. 10 “Well, mine’s a … he says.” - Par. 20 “I was seven when … trying to keep up.” - Par. 22 “Finally we had climbed … like turned-up soil.” - Par. 25 “Gale was trying … When he knew.” | Chapter Sara 2002  - Par. 144 “When Kate goes into the hospital … what was best for you” |
| Number of words | 628 | 813 | 702 | 539 |
| Reading time^b^ | 2:35 min. | 3:31 min. | 2:23 min. | 1:58 min. |
| Mood^b^ | Happy  (*M* = 4, *SD* = 1.51) | Happy  (*M* = 3.75, *SD* = 0.7) | Sad  (*M* = -4.38, *SD* = 0.74) | Sad  (*M* = -4.5, *SD* = 0.54) |

^b^Reading time and Emotional mood were assessed in the pretest (Emotional mood: -5 = sad, 0 = neutral, +5 = happy)
