# Supplementary material for: Emotionally congruent music and text increase immersion and appraisal
Source: PLoS One. 2023 Jan 12;18(1):e0280019. doi: 10.1371/journal.pone.0280019 (PMC9836297; doi:10.1371/journal.pone.0280019)
Supplement: S2 Table — (DOCX) [file pone.0280019.s002.docx]

**S2 Table. List of music pieces presented as audio stimuli.**

| Composer and Title | Antonio Vivaldi: The four seasons, ‘Spring’, Allegro | Wolfgang Amadeus Mozart: Serenade No. 13, K 525, ‘Eine Kleine Nachtmusik' - Allegro | Edvard Grieg: Peer Gynt Suite No. 1, Op. 46, ‘The Death of Åse’ | Tomaso Albinoni: Adagio for Strings |
| --- | --- | --- | --- | --- |
| Artist | Unknown | Baroque Orchestra ‘Tafelmusik’ | BBC Scottish Symphony Orchestra | Hamburg Chamber Orchestra |
| Excerpt | 3 x 0:00 - 1:27 min.  + 1 x 0:00 - 01:05 min. | 2 x 0:05 - 02:51 min.  + 1 x 0:00 + 01:09 min. | 2 x 0:00 - 1:36 min.  + 1 x 0:00 - 0:47 min. | 2 x 0:00 – 1:52 min. + 1 x 0:00 – 0:15 |
| BPM^c^ | 100 | 150 | 42 | 47 |
| Instruments | String ensemble, harpsichord | String ensemble | String orchestra | String orchestra, organ |
| Key | E major | G major | B minor | G minor |
| Mood^b^ | Happy  (*M* = 4.2, SD = 0.73) | Happy  (*M* = 3.98, SD = 1.08) | Sad  (*M* = -2.58, SD = 1.27) | Sad  (*M* = -2.76, SD = 1.72) |
| Familiarity^b^ | *M* = 8.09, SD = 1.9 | *M* = 8.36, SD = 1.37 | *M* = 1.78, SD = 2.15 | *M* = 2.07, SD = 2.25 |
| Studies employing the stimuli to induce happy or sad mood | [44, 89–91] | [89, 92–94] | [95–98] | [44, 89, 90, 92] |

^c^Beats per minute averaged over 30 seconds; ^b^Familiarity and Emotional mood were assessed in the pretest (Familiarity: 0 = ‘not familiar at all’, 9 = ‘very familiar’; Emotional mood: -5 = sad, 0 = neutral, +5 = happy)
