# Supplementary material for: Emotionally congruent music and text increase immersion and appraisal
Source: PLoS One. 2023 Jan 12;18(1):e0280019. doi: 10.1371/journal.pone.0280019 (PMC9836297; doi:10.1371/journal.pone.0280019)
Supplement: S3 Table — (DOCX) [file pone.0280019.s003.docx]

**S3 Table. Pearson’s correlation coefficients of valence, arousal, and dominance experienced by the participants with perceived text-mood score, text liking, and text immersion.**

| Pearson’s r | | Text-mood score | | | | Text liking | | | | Text immersion | | | |
| --- | --- | --- | --- | --- | --- | --- | --- | --- | --- | --- | --- | --- | --- |
|  |  | HMHT^e^ | HMST^f^ | SMHT^g^ | SMST^h^ | HMHT | HMST | SMHT | SMST | HMHT | HMST | SMHT | SMST |
| Δ-valence^d^ | HMHT | .137 | .041 | -.035 | .260 | .199 | -.002 | .128 | -.006 | .357* | .024 | -.039 | -.103 |
|  | HMST | -.061 | .073 | -.068 | .040 | -.294 | .265 | -.281 | -.055 | -.261 | -.398* | -.379* | -.179 |
|  | SMHT | .059 | -.302 | .323* | -.148 | .110 | -.117 | .068 | .030 | .180 | .061 | -.005 | .249 |
|  | SMST | -.311 | .065 | -.084 | .236 | -.421** | .133 | -.496** | .001 | -.436** | -.273 | -.412** | -.372* |
| Δ-arousal^d^ | HMHT | .056 | .038 | -.003 | .168 | .113 | .073 | .005 | -.099 | .170 | -.215 | .052 | -.103 |
|  | HMST | .270 | .264 | -.086 | .365* | .284 | -.015 | .179 | .074 | .257 | .074 | .136 | -.115 |
|  | SMHT | -.222 | .212 | -.163 | .275 | -.124 | .190 | -.070 | -.016 | -.063 | -.199 | .046 | -.225 |
|  | SMST | .243 | .181 | -.046 | .181 | .201 | .223 | .166 | .065 | .265 | .035 | .125 | .095 |
| Δ-dominance^d^ | HMHT | .066 | -.309 | .285 | -.291 | .080 | -.030 | .133 | -.062 | .044 | ,398* | .097 | .265 |
|  | HMST | .128 | .068 | -.047 | -.151 | .142 | -.166 | .371* | .028 | .087 | .171 | ,431** | .183 |
|  | SMHT | .091 | -.162 | .035 | -.208 | .039 | .011 | .124 | -.051 | -.061 | .303 | .224 | .172 |
|  | SMST | .377* | -.095 | .039 | -.273 | .060 | -.265 | .169 | .025 | .030 | .189 | .179 | .248 |

^d^Δ-values refer to differences from the baseline measurement at the beginning of the experiment. ^e^happy-music-happy-text combinations, ^f^happy-music-sad-text combinations, ^g^sad-music-happy-text combinations, ^h^sad-music-sad-text combinations. Asterisks indicate significant effects (*: *p* < .05; **: *p* < .01).
