# Supplementary material for: Emotionally congruent music and text increase immersion and appraisal
Source: PLoS One. 2023 Jan 12;18(1):e0280019. doi: 10.1371/journal.pone.0280019 (PMC9836297; doi:10.1371/journal.pone.0280019)
Supplement: S4 Table — (DOCX) [file pone.0280019.s004.docx]

**S4 Table. Pearson’s correlation coefficients of valence, arousal, and dominance experienced by the subjects with perceived music-mood score, music liking, and music immersion**

|  |  | Music-mood score | | | | Music liking | | | | Music immersion | | | |
| --- | --- | --- | --- | --- | --- | --- | --- | --- | --- | --- | --- | --- | --- |
|  |  | HMHT^e^ | HMST^f^ | SMHT^g^ | SMST^h^ | HMHT | HMST | SMHT | SMST | HMHT | HMST | SMHT | SMST |
| Δ-valence^d^ | HMHT | -.061 | .067 | -.038 | -.004 | .088 | .016 | .025 | .057 | -.021 | .134 | .080 | .097 |
|  | HMST | .000 | .080 | -.024 | -.056 | -.011 | .049 | -.120 | -.145 | .057 | .171 | -.152 | -.215 |
|  | SMHT | -.127 | .236 | .185 | .092 | -.203 | -.116 | -.234 | -.107 | -.037 | -.045 | -.262 | -.101 |
|  | SMST | -.139 | -.057 | .068 | .199 | -.145 | -.113 | -.332* | -.258 | .010 | -.142 | -.400* | -.467** |
| Δ-arousal^d^ | HMHT | -.015 | -.013 | .148 | .046 | .032 | .114 | .226 | .060 | .070 | .242 | -.129 | -.034 |
|  | HMST | .056 | -.261 | -.107 | -.043 | .306 | .133 | .322* | .198 | .048 | .234 | .308 | .180 |
|  | SMHT | -.071 | -.181 | .032 | .352* | -.077 | .083 | -.030 | -.003 | -.040 | .087 | -.091 | -.167 |
|  | SMST | .266 | -.018 | -.030 | -.078 | .399* | .438** | .428** | .330* | .136 | .369* | .106 | .073 |
| Δ-dominance^d^ | HMHT | .262 | .299 | -.167 | -.270 | -.152 | -.124 | -.214 | -.288 | -.277 | -.265 | -.030 | -.088 |
|  | HMST | .054 | -.128 | -.101 | -.115 | -.010 | -.203 | .071 | -.014 | -.073 | -.131 | .334* | .216 |
|  | SMHT | .033 | -.086 | -.123 | -.076 | -.145 | -.136 | -.039 | -.243 | -.135 | -.103 | .054 | -.129 |
|  | SMST | .108 | .168 | -.005 | -.182 | .158 | .033 | .040 | .030 | -.083 | .045 | .135 | .204 |

^d^Δ-values refer to differences from the baseline measurement at the beginning of the experiment. ^e^happy-music-happy-text combinations, ^f^happy-music-sad-text combinations, ^g^sad-music-happy-text combinations, ^h^sad-music-sad-text combinations. Asterisks indicate significant effects (*: *p* < .05; **: *p* < .01).
