# Supplementary material for: Emotionally congruent music and text increase immersion and appraisal
Source: PLoS One. 2023 Jan 12;18(1):e0280019. doi: 10.1371/journal.pone.0280019 (PMC9836297; doi:10.1371/journal.pone.0280019)
Supplement: S5 Table — (DOCX) [file pone.0280019.s005.docx]

**S5 Table. Results of a 2 (music category) x 2 (text category) rmANOVA on music-text fit.**

| Music or text dimensions | *F* | df | *p* | η² |
| --- | --- | --- | --- | --- |
| Music category | 48.43** | 1 / 39 | <.001 | .554 |
| Text category | 0.01 | 1 / 39 | .944 | .000 |
| Music category x text category | 111.55** | 1 / 39 | <.001 | .741 |

Asterisks indicate significant effects (*: *p* < .05; **: *p* < .01).
