# Supplementary material for: Emotionally congruent music and text increase immersion and appraisal
Source: PLoS One. 2023 Jan 12;18(1):e0280019. doi: 10.1371/journal.pone.0280019 (PMC9836297; doi:10.1371/journal.pone.0280019)
Supplement: S6 Table — (DOCX) [file pone.0280019.s006.docx]

**S6 Table. Multivariate effects and interactions of a 2 (music category) x 2 (text category) rmMANOVA with the between-subjects factor musical expertise on perceived mood score, quality, immersion, and liking of the text.**

| Music or text dimensions | *F* | df | *p* | η² |
| --- | --- | --- | --- | --- |
| Music category | 2.81* | 4 / 35 | .040 | .243 |
| Music category x musical expertise | 0.24 | 4 / 35 | .913 | .027 |
| Text category | 70.65** | 4 / 35 | <.001 | .890 |
| Text category x musical expertise | 1.08 | 4 / 35 | .382 | .110 |
| Music category x text category | 3.63* | 4 / 35 | .014 | .293 |
| Music category x text category x musical expertise | .29* | 4 / 35 | .885 | .032 |

Effects refer to Pillai’s trace values. Asterisks indicate significant effects (*: *p* < .05; **: *p* < .01).
