# Supplementary material for: Emotionally congruent music and text increase immersion and appraisal
Source: PLoS One. 2023 Jan 12;18(1):e0280019. doi: 10.1371/journal.pone.0280019 (PMC9836297; doi:10.1371/journal.pone.0280019)
Supplement: S7 Table — (DOCX) [file pone.0280019.s007.docx]

**S7 Table. Multivariate effects and interactions of a 2 (music category) x 2 (text category) rmMANOVA with the between-subjects factor reading frequency on perceived mood score, quality, immersion, and liking of the music.**

| Music or text dimensions | *F* | df | *p* | η² |
| --- | --- | --- | --- | --- |
| Music category | 49.71** | 4 / 35 | <.001 | .854 |
| Music category x reading frequency | 1.00 | 4 / 35 | .420 | .105 |
| Text category | 4.86** | 4 / 35 | .003 | .364 |
| Text category x reading frequency | 0.41 | 4 / 35 | .804 | .045 |
| Music category x text category | 3.65* | 4 / 35 | .014 | .301 |
| Music category x text category x reading frequency | 0.21 | 4 / 35 | .932 | .024 |

Effects refer to Pillai’s trace values. Asterisks indicate significant effects (*: *p* < .05; **: *p* < .01).
