# Supplementary material for: Emotionally congruent music and text increase immersion and appraisal
Source: PLoS One. 2023 Jan 12;18(1):e0280019. doi: 10.1371/journal.pone.0280019 (PMC9836297; doi:10.1371/journal.pone.0280019)
Supplement: S8 Table — (DOCX) [file pone.0280019.s008.docx]

**S8 Table. Multivariate effects and interactions of a 2 (music category) x 2 (text category) rmMANOVA with the between-subjects factor ‘habit to listen to music while reading’ on perceived mood score, quality, immersion, and liking of the text.**

| Music or text dimensions | | *F* | df | *p* | η² |
| --- | --- | --- | --- | --- | --- |
| **Music category** | | **3.15*** | **4 / 35** | **.026** | **.265** |
| **Music category x habit** | | **0.74** | **4 / 35** | **.570** | **.078** |
| **Text category** | | **39.5**** | **4 / 35** | **<.001** | **.819** |
| **Text category x habit** | | **0.19** | **4 / 35** | **.941** | **.021** |
| **Music category x text category** | | **3.23*** | **4 / 35** | **.024** | **.269** |
| **Music category x text category x habit** | | **0.83** | **4 / 35** | **.518** | **.086** |
| **Between-subjects factor ‘habit’** | |  |  |  |  |
|  | Text-mood score | 5.53* | 1 / 38 | .024 | .127 |
|  | Text quality | 0.51 | 1 / 38 | .48 | .013 |
|  | Text immersion | 0.32 | 1 / 38 | .575 | .008 |
|  | Text liking | 0.29 | 1 / 38 | .592 | .008 |

Effects refer to Pillai’s trace values. Asterisks indicate significant effects (*: *p* < .05; **: *p* < .01**).**
