# Supplementary material for: Emotionally congruent music and text increase immersion and appraisal
Source: PLoS One. 2023 Jan 12;18(1):e0280019. doi: 10.1371/journal.pone.0280019 (PMC9836297; doi:10.1371/journal.pone.0280019)
Supplement: S1 Questionnaire — To evaluate music and text for the group “music first”. (PDF) [file pone.0280019.s009.pdf]

EM MUSIC 1 **VP-No.___M___**

M1.1. How do you feel right now? Please mark the corresponding male or a space in between.

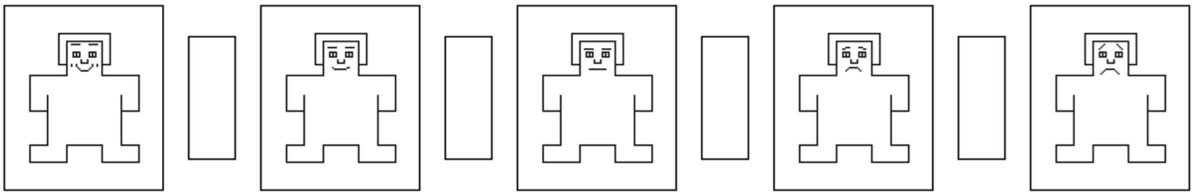

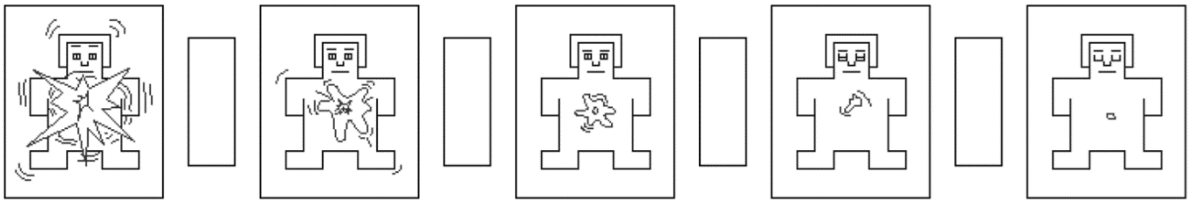

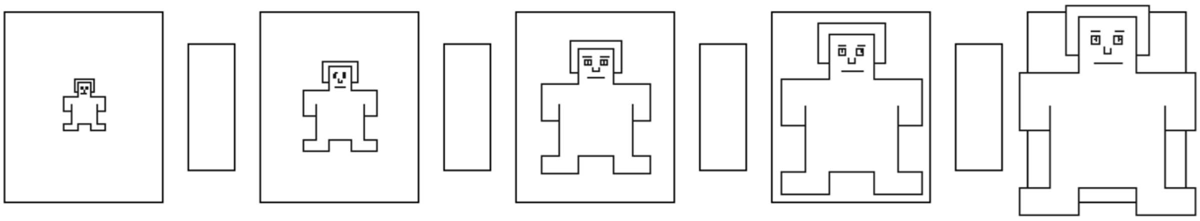

Please tick the number that applies:

M1.2. How do you like the music?

0	1	2	3	4	5	6	7	8	9
Not at all									Very much

M1.3. How artistically valuable is the music?

0	1	2	3	4	5	6	7	8	9
Not at all									Very much

M1.4. how much did the music involve you?

0	1	2	3	4	5	6	7	8	9
Not at all									Very much

M1.5. How familiar were you with the music before this survey?

0	1	2	3	4	5	6	7	8	9
Not at all									Very much

M1.6a. What was the mood of the music?

-5	-4	-3	-2	-1	0	1	2	3	4	5
Sad					Neutral					Happy

M1.6b. How strong was this mood?

0	1	2	3	4	5	6	7	8	9
Not at all									Very much

TEXT 1 **VP-No.___ T____**

T1.1. How much do you like the text?

0	1	2	3	4	5	6	7	8	9
Not at all									Very much

T1.2. How artistically valuable is the text?

0	1	2	3	4	5	6	7	8	9
Not at all									Very much

T1.3. How much did the text involve you?

0	1	2	3	4	5	6	7	8	9
Not at all									Very much

T1.4a. What was the emotional mood of the text?

-5	-4	-3	-2	-1	0	1	2	3	4	5
Sad					Neutral					Happy

T1.4b. How strong was this mood?

0	1	2	3	4	5	6	7	8	9
Not at all									Very much

TEXT-MUSIC-FIT 1

TM1.1. How well did the text and music fit together in your perception?

0	1	2	3	4	5	6	7	8	9
Not at all									Very much

EM MUSIC 2 **VP-No.___M___**

M2.1. How do you feel right now? Please mark the corresponding male or a space in between.

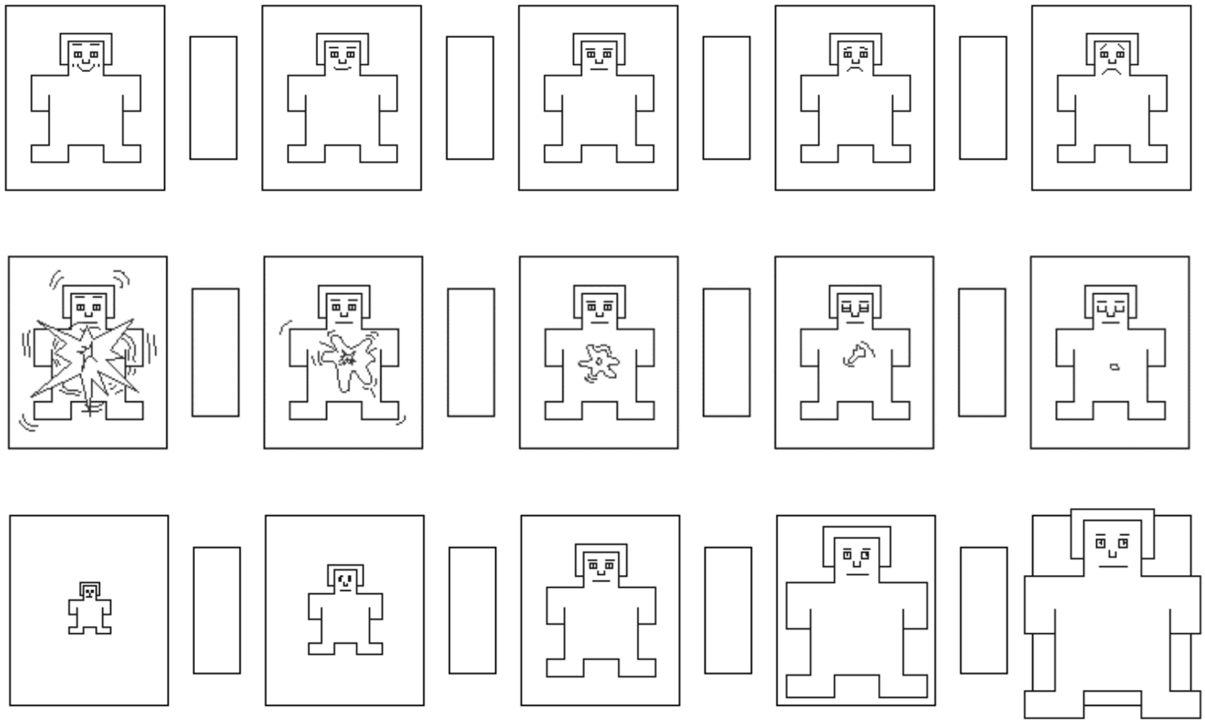

Please tick the number that applies:

M2.2. How do you like the music?

0	1	2	3	4	5	6	7	8	9
Not at All									Very much

M2.3. How artistically valuable is the music?

0	1	2	3	4	5	6	7	8	9
Not at All									Very much

M2.4. how much did the music involve you?

0	1	2	3	4	5	6	7	8	9
Not at All									Very much

M2.5. How familiar were you with the music before this survey?

0	1	2	3	4	5	6	7	8	9
Not at All									Very much

M2.6a. What was the mood of the music?

-5	-4	-3	-2	-1	0	1	2	3	4	5
Sad					Neutral					Happy

M2.6b. How strong was this mood?

0	1	2	3	4	5	6	7	8	9
Not at All									Very much

TEXT 2 **VP-No.___** **T____**

T2.1. How much do you like the text?

0	1	2	3	4	5	6	7	8	9
Not at all									Very much

T2.2. How artistically valuable is the text?

0	1	2	3	4	5	6	7	8	9
Not at all									Very much

T2.3. How much did the text involve you?

0	1	2	3	4	5	6	7	8	9
Not at all									Very much

T2.4a. What was the emotional mood of the text?

-5	-4	-3	-2	-1	0	1	2	3	4	5
Sad					Neutral					Happy

T2.4b. How strong was this mood?

0	1	2	3	4	5	6	7	8	9
Not at all									Very much

TEXT-MUSIC-FIT 2

TM2.1. How well did the text and music fit together in your perception?

0	1	2	3	4	5	6	7	8	9
Not at all									Very much

EM MUSIC 3 **VP-No.___M___**

M3.1. How do you feel right now? Please mark the corresponding male or a space in between.

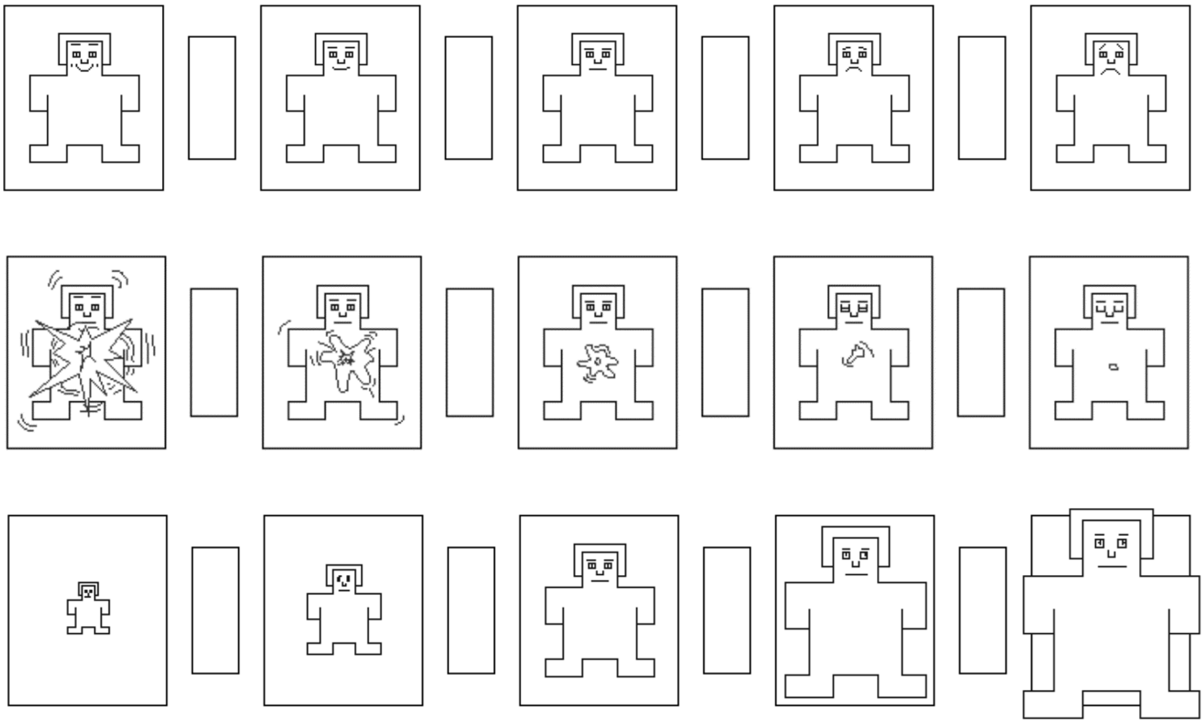

Please tick the number that applies:

M3.2. How do you like the music?

0	1	2	3	4	5	6	7	8	9
Not at all									Very much

M3.3. How artistically valuable is the music?

0	1	2	3	4	5	6	7	8	9
Not at all									Very much

M3.4. how much did the music involve you?

0	1	2	3	4	5	6	7	8	9
Not at all									Very much

M3.5. How familiar were you with the music before this survey?

0	1	2	3	4	5	6	7	8	9
Not at all									Very much

M3.6a. What was the mood of the music?

-5	-4	-3	-2	-1	0	1	2	3	4	5
Sad					Neutral					Happy

M3.6b. How strong was this mood?

0	1	2	3	4	5	6	7	8	9
Not at all									Very much

TEXT 1 **VP-No.___ T____**

T3.1. How much do you like the text?

0	1	2	3	4	5	6	7	8	9
Not at all									Very much

T3.2. How artistically valuable is the text?

0	1	2	3	4	5	6	7	8	9
Not at all									Very much

T3.3. How much did the text involve you?

0	1	2	3	4	5	6	7	8	9
Not at all									Very much

T3.4a. What was the emotional mood of the text?

-5	-4	-3	-2	-1	0	1	2	3	4	5
Sad					Neutral					Happy

T3.4b. How strong was this mood?

0	1	2	3	4	5	6	7	8	9
Not at all									Very much

TEXT-MUSIC-FIT 1

TM3.1. How well did the text and music fit together in your perception?

0	1	2	3	4	5	6	7	8	9
Not at all									Very much

EM MUSIC 4 **VP-No.___M___**

M4.1. How do you feel right now? Please mark the corresponding male or a space in between.

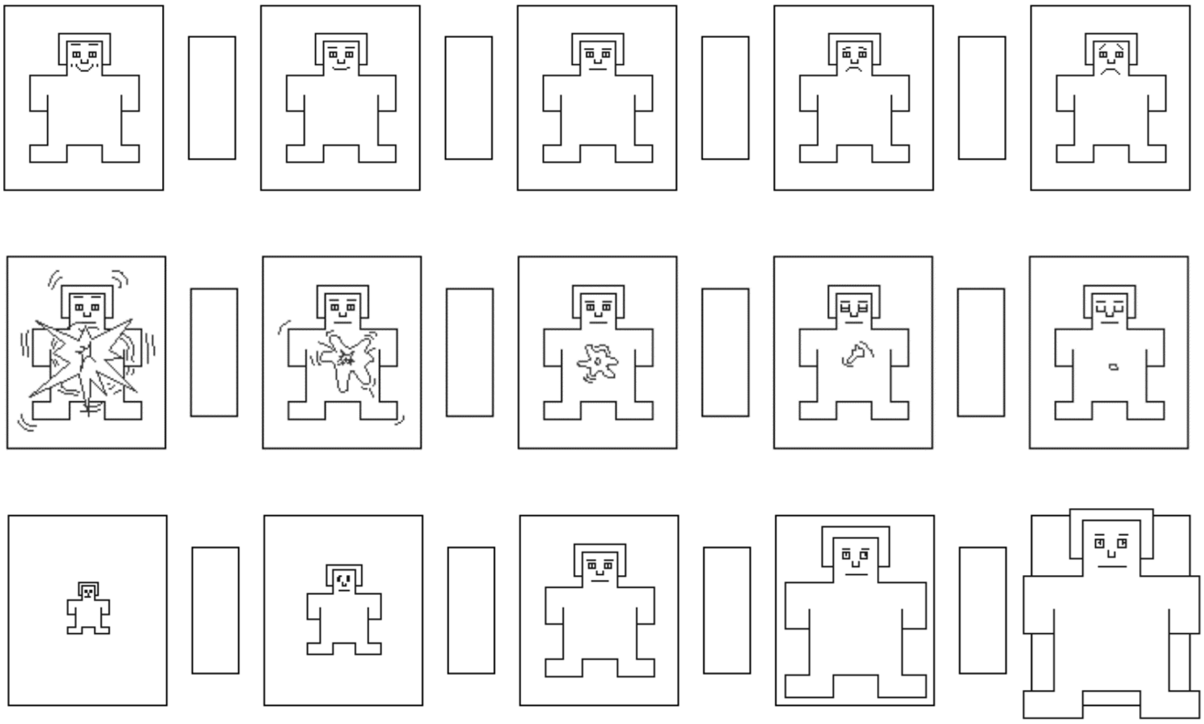

Please tick the number that applies:

M4.2. How do you like the music?

0	1	2	3	4	5	6	7	8	9
Not at all									Very much

M4.3. How artistically valuable is the music?

0	1	2	3	4	5	6	7	8	9
Not at all									Very much

M4.4. how much did the music involve you?

0	1	2	3	4	5	6	7	8	9
Not at all									Very much

M4.5. How familiar were you with the music before this survey?

0	1	2	3	4	5	6	7	8	9
Not at all									Very much

M4.6a. What was the mood of the music?

-5	-4	-3	-2	-1	0	1	2	3	4	5
Sad					Neutral					Happy

M4.6b. How strong was this mood?

0	1	2	3	4	5	6	7	8	9
Not at all									Very much

TEXT 4 **VP-No.___ T____**

T4.1. How much do you like the text?

0	1	2	3	4	5	6	7	8	9
Not at all									Very much

T4.2. How artistically valuable is the text?

0	1	2	3	4	5	6	7	8	9
Not at all									Very much

T4.3. How much did the text involve you?

0	1	2	3	4	5	6	7	8	9
Not at all									Very much

T4.4a. What was the emotional mood of the text?

-5	-4	-3	-2	-1	0	1	2	3	4	5
Sad					Neutral					Happy

T4.4b. How strong was this mood?

0	1	2	3	4	5	6	7	8	9
Not at all									Very much

TEXT-MUSIC-FIT 1

TM4.1. How well did the text and music fit together in your perception?

0	1	2	3	4	5	6	7	8	9
Not at all									Very much
