# Supplementary material for: Emotionally congruent music and text increase immersion and appraisal
Source: PLoS One. 2023 Jan 12;18(1):e0280019. doi: 10.1371/journal.pone.0280019 (PMC9836297; doi:10.1371/journal.pone.0280019)
Supplement: S2 Questionnaire — To evaluate music and text for the group “text first”. (PDF) [file pone.0280019.s010.pdf]

ET TEXT 1 **VP-No.___T___**

T1.1 How do you feel right now? Please mark the corresponding manikin or a space in between.

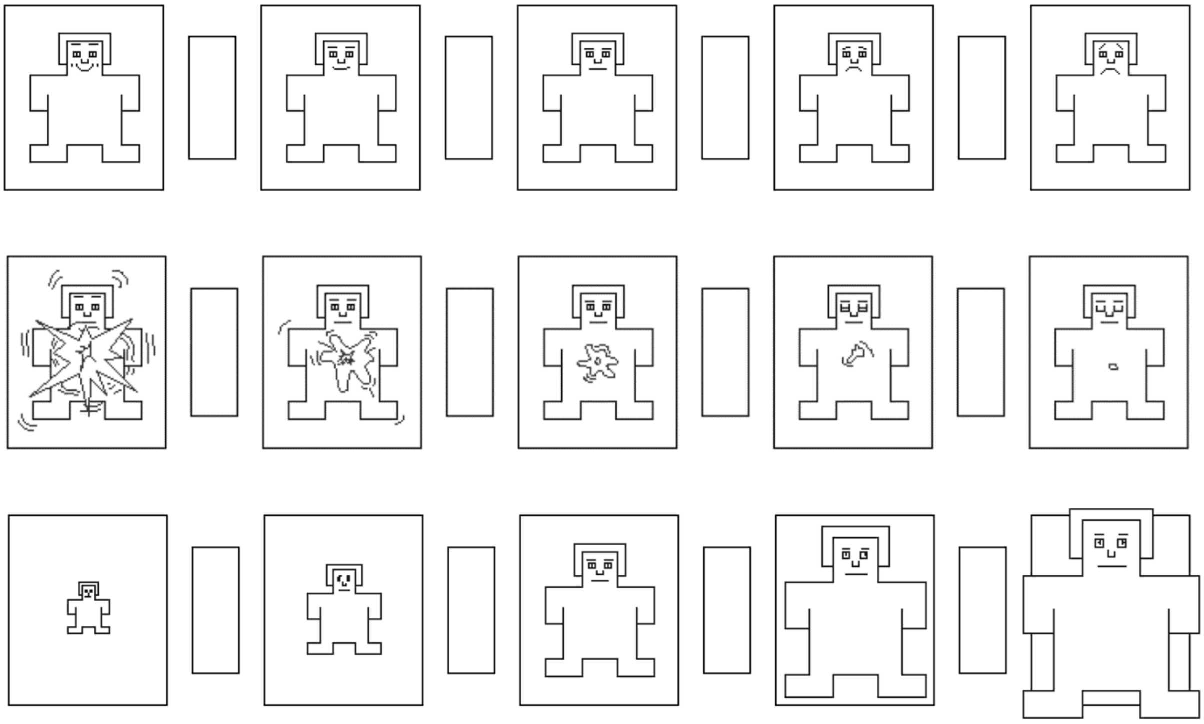

Please tick the number that applies:

T1.2 How much do you like the text?

0	1	2	3	4	5	6	7	8	9
Not at all									Very much

T1.3 How artistically valuable is the text?

0	1	2	3	4	5	6	7	8	9
Not at all									Very much

T1.4 How much did the text involve you?

0	1	2	3	4	5	6	7	8	9
Not at all									Very much

T1.5a What was the emotional mood of the text?

-5	-4	-3	-2	-1	0	1	2	3	4	5
Sad					Neutral					Happy

T1.5b How strong was this mood?

0	1	2	3	4	5	6	7	8	9
Not at all									Very much

MUSIC 1 VP-Nr.___ M____

M1.2. How do you like the music?

0	1	2	3	4	5	6	7	8	9
Not at all									Very much

M1.3. How artistically valuable is the music?

0	1	2	3	4	5	6	7	8	9
Not at all									Very much

M1.4. how much did the music involve you?

0	1	2	3	4	5	6	7	8	9
Not at all									Very much

M1.5. How familiar were you with the music before this survey?

0	1	2	3	4	5	6	7	8	9
Not at all									Very much

M1.6a. What was the mood of the music?

-5	-4	-3	-2	-1	0	1	2	3	4	5
Sad					Neutral					Happy

TEXT-MUSIC-FIT 1

TM1.1. How well did the text and music fit together in your perception?

0	1	2	3	4	5	6	7	8	9
Not at all									Very much

ET TEXT 2 **VP-No.___T___**

T2.1 How do you feel right now? Please mark the corresponding manikin or a space in between.

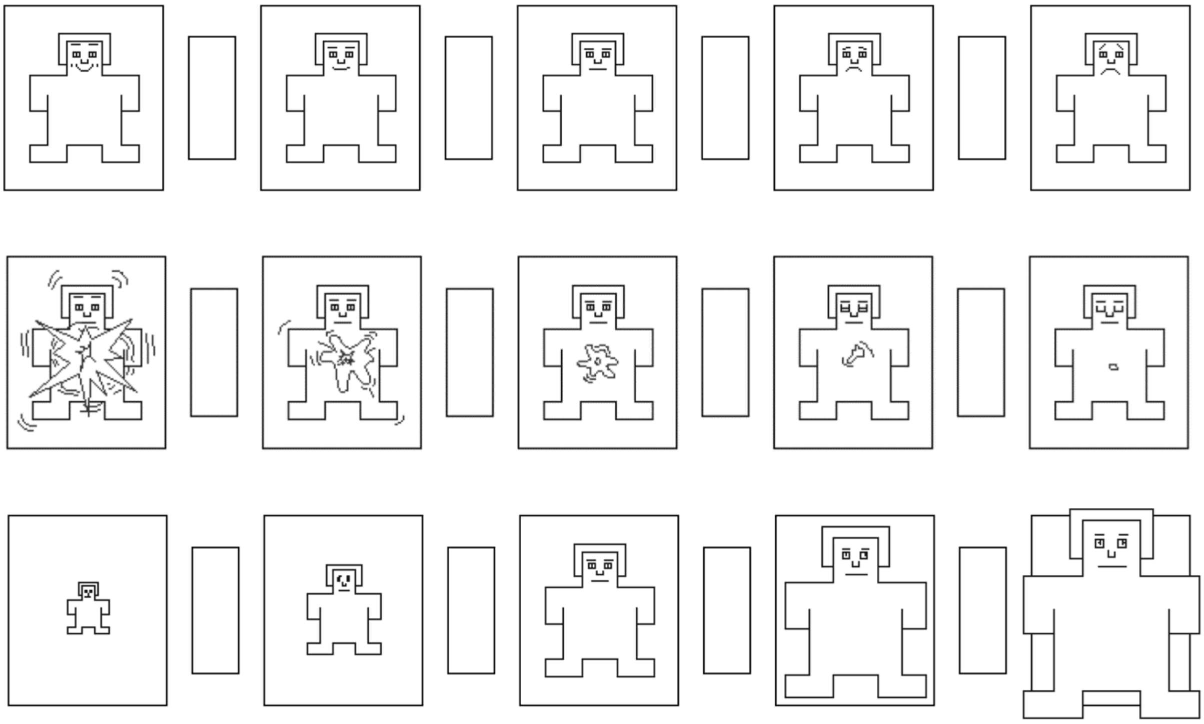

Please tick the number that applies:

T2.2 How much do you like the text?

0	1	2	3	4	5	6	7	8	9
Not at all									Very much

T2.3 How artistically valuable is the text?

0	1	2	3	4	5	6	7	8	9
Not at all									Very much

T2.4 How much did the text involve you?

0	1	2	3	4	5	6	7	8	9
Not at all									Very much

T2.5a What was the emotional mood of the text?

-5	-4	-3	-2	-1	0	1	2	3	4	5
Sad					Neutral					Happy

T2.5b How strong was this mood?

0	1	2	3	4	5	6	7	8	9
Not at all									Very much

MUSIC 2 VP-Nr.___ M____

M2.2. How do you like the music?

0	1	2	3	4	5	6	7	8	9
Not at all									Very much

M2.3. How artistically valuable is the music?

0	1	2	3	4	5	6	7	8	9
Not at all									Very much

M2.4. how much did the music involve you?

0	1	2	3	4	5	6	7	8	9
Not at all									Very much

M2.5. How familiar were you with the music before this survey?

0	1	2	3	4	5	6	7	8	9
Not at all									Very much

M2.6a. What was the mood of the music?

-5	-4	-3	-2	-1	0	1	2	3	4	5
Sad					Neutral					Happy

TEXT-MUSIC-FIT 2

TM2.1. How well did the text and music fit together in your perception?

0	1	2	3	4	5	6	7	8	9
Not at all									Very much

ET TEXT 3 **VP-No.___T___**

T3.1 How do you feel right now? Please mark the corresponding manikin or a space in between.

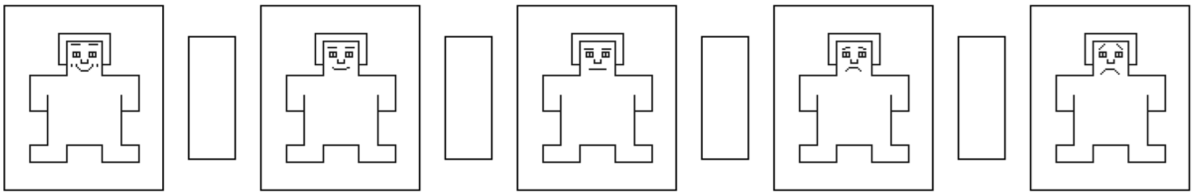

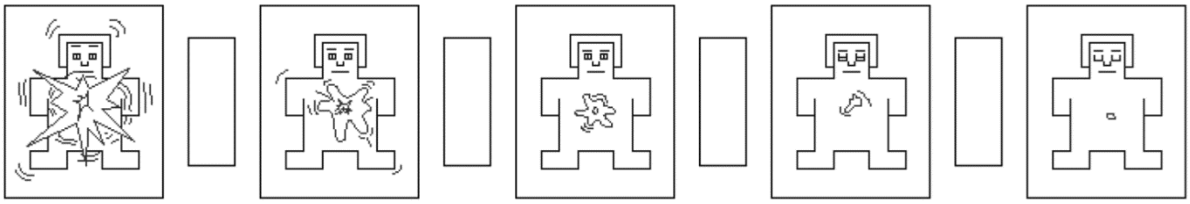

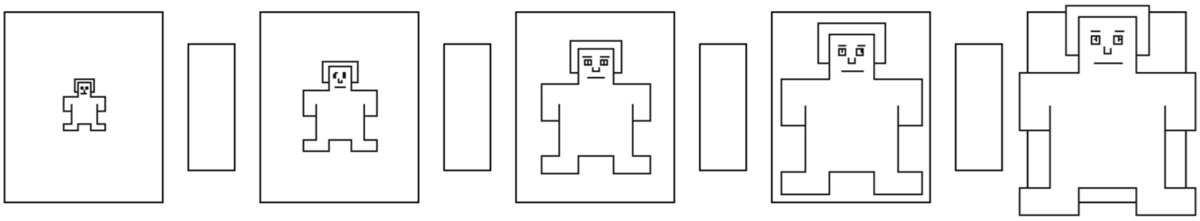

Please tick the number that applies:

T3.2 How much do you like the text?

0	1	2	3	4	5	6	7	8	9
Not at all									Very much

T3.3 How artistically valuable is the text?

0	1	2	3	4	5	6	7	8	9
Not at all									Very much

T3.4 How much did the text involve you?

0	1	2	3	4	5	6	7	8	9
Not at all									Very much

T3.5a What was the emotional mood of the text?

-5	-4	-3	-2	-1	0	1	2	3	4	5
Sad					Neutral					Happy

T3.5b How strong was this mood?

0	1	2	3	4	5	6	7	8	9
Not at all									Very much

MUSIC 3 VP-Nr.___ M____

M3.2. How do you like the music?

0	1	2	3	4	5	6	7	8	9
Not at all									Very much

M3.3. How artistically valuable is the music?

0	1	2	3	4	5	6	7	8	9
Not at all									Very much

M3.4. how much did the music involve you?

0	1	2	3	4	5	6	7	8	9
Not at all									Very much

M3.5. How familiar were you with the music before this survey?

0	1	2	3	4	5	6	7	8	9
Not at all									Very much

M3.6a. What was the mood of the music?

-5	-4	-3	-2	-1	0	1	2	3	4	5
Sad					Neutral					Happy

TEXT-MUSIC-FIT 3

TM3.1. How well did the text and music fit together in your perception?

0	1	2	3	4	5	6	7	8	9
Not at all									Very much

ET TEXT 4 **VP-No.___T___**

T4.1 How do you feel right now? Please mark the corresponding manikin or a space in between.

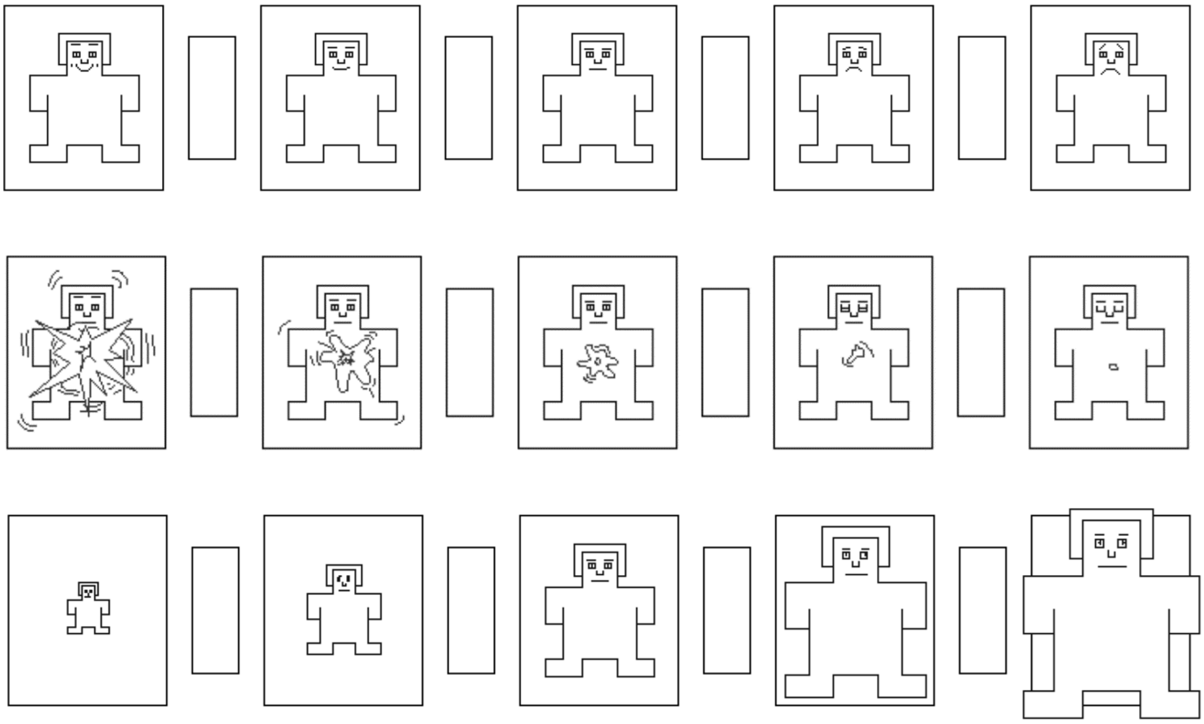

Please tick the number that applies:

T4.2 How much do you like the text?

0	1	2	3	4	5	6	7	8	9
Not at all									Very much

T4.3 How artistically valuable is the text?

0	1	2	3	4	5	6	7	8	9
Not at all									Very much

T4.4 How much did the text involve you?

0	1	2	3	4	5	6	7	8	9
Not at all									Very much

T4.5a What was the emotional mood of the text?

-5	-4	-3	-2	-1	0	1	2	3	4	5
Sad					Neutral					Happy

T4.5b How strong was this mood?

0	1	2	3	4	5	6	7	8	9
Not at all									Very much

MUSIC 4

VP-Nr.___ M____

M4.2. How do you like the music?

0	1	2	3	4	5	6	7	8	9
Not at all									Very much

M4.3. How artistically valuable is the music?

0	1	2	3	4	5	6	7	8	9
Not at all									Very much

M4.4. how much did the music involve you?

0	1	2	3	4	5	6	7	8	9
Not at all									Very much

M4.5. How familiar were you with the music before this survey?

0	1	2	3	4	5	6	7	8	9
Not at all									Very much

M4.6a. What was the mood of the music?

-5	-4	-3	-2	-1	0	1	2	3	4	5
Sad					Neutral					Happy

TEXT-MUSIC-FIT 4

TM4.1. How well did the text and music fit together in your perception?

0	1	2	3	4	5	6	7	8	9
Not at all									Very much
