# Supplementary material for: Emotionally congruent music and text increase immersion and appraisal
Source: PLoS One. 2023 Jan 12;18(1):e0280019. doi: 10.1371/journal.pone.0280019 (PMC9836297; doi:10.1371/journal.pone.0280019)
Supplement: S3 Questionnaire — To assess music listening and reading behavior and demographic data. (PDF) [file pone.0280019.s011.pdf]

Follow-up interview **VP-No.___**

1. Do you have a known visual impairment? □ Yes □ No

   1a. If yes, which? ________________________________

   1b. If yes, do you wear any vision aids? □ Yes □ No

2. Do you have a known hearing loss? □ Yes □ No

   2a. If yes, which? ________________________________

   2b. If yes, do you wear a hearing aid? □ Yes □ No

3. Are you a synesthete? □ Yes □ No

   3a. If yes, what type of synesthesia? ________________________________

4. Gender □ M □ F □ D

5. Age _______

6. Profession
   - □ Psychology student
   - □ Non-psychology student
   - □ psychologist
   - □ musician
   - □ other profession ________________________________

7. highest level of education
   - □ None
   - □ Advanced school leaving certificate
   - □ Secondary school certificate
   - □ High school degree
   - □ Completed apprenticeship
   - □ Bachelor's degree
   - □ Master’s degree / Diploma
   - □ PhD
   - □ Habilitation

	(almost) never	Several times per month	Several times per week	(almost) daily
8. How often do you read for pleasure (books, newspapers, magazines, news texts; analog or digital)?	□ 1	□ 2	□ 3	□ 4
9. as a child, how often did you read for pleasure?	□ 1	□ 2	□ 3	□ 4
10. how often do you listen to music for pleasure?	□ 1	□ 2	□ 3	□ 4
11. as a child, how often did you listen to music for pleasure?	□ 1	□ 2	□ 3	□ 4

12. why do you listen to music?
    - □ to relax
    - □ to pass the time
    - □ because it reminds me of something
    - □ because I want to create a certain image
    - □ because I am interested in the music itself
    - □ as background music
    - □ other ______________________
    - □ for fun
    - □ because it is healthy
    - □ because I like to hear the lyrics
    - □ because I want to influence my emotions
    - □ because it is difficult to avoid it
    - □ to create a nice atmosphere with friends
    - □ I don't listen to music.

13. when you listen to music in connection with other activities, what are they?
    - □ when waking up
    - □ while doing sports
    - □ while doing housework
    - □ when eating
    - □ as background music for dating
    - □ when going to bed / to fall asleep
    - □ on the train, bus or plane
    - □ I do not listen to music.
    - □ when taking a bath
    - □ while working / studying
    - □ while resting
    - □ as background music with friends
    - □ while reading
    - □ when riding a bike or driving a car
    - □ other ____________________

**Please continue to relate the following questions to Reading for pleasure / in leisure time** (Books, newspapers, magazines, news texts; analog or digital)	Do not agree at all	Do not agree	Do rather not agree	neither agree nor disagree	Rather agree	agree	Fully agree
14. I only read when I have to.	□ 1	□ 2	□ 3	□ 4	□ 5	□ 6	□ 7
15. I like to talk with other people about things I have read.	□ 1	□ 2	□ 3	□ 4	□ 5	□ 6	□ 7
16. I like to spend my free time reading.	□ 1	□ 2	□ 3	□ 4	□ 5	□ 6	□ 7
17. I read only to get information.	□ 1	□ 2	□ 3	□ 4	□ 5	□ 6	□ 7
18. Reading is an important activity at my home.	□ 1	□ 2	□ 3	□ 4	□ 5	□ 6	□ 7
19. reading is a kind of addiction for me - I couldn't live without books and newspapers.	□ 1	□ 2	□ 3	□ 4	□ 5	□ 6	□ 7
20. In the digital age, reading is no longer so important.	□ 1	□ 2	□ 3	□ 4	□ 5	□ 6	□ 7
21. I would not describe myself as a reader.	□ 1	□ 2	□ 3	□ 4	□ 5	□ 6	□ 7
22. Reading is part of my daily routine.	□ 1	□ 2	□ 3	□ 4	□ 5	□ 6	□ 7
23. i find reading exhausting.	□ 1	□ 2	□ 3	□ 4	□ 5	□ 6	□ 7
24. I like to listen to music while reading.	□ 1	□ 2	□ 3	□ 4	□ 5	□ 6	□ 7
25. I often read when I am out and about.	□ 1	□ 2	□ 3	□ 4	□ 5	□ 6	□ 7
26. I read mostly on my cell phone/tablet.	□ 1	□ 2	□ 3	□ 4	□ 5	□ 6	□ 7
27. I only read when I really have a lot of time.	□ 1	□ 2	□ 3	□ 4	□ 5	□ 6	□ 7

	Do not agree at all	Do not agree	Do rather not agree	neither agree nor disagree	Rather agree	agree	Fully agree
28. when someone else sings a song that I don't know, I can usually sing along.	□ 1	□ 2	□ 3	□ 4	□ 5	□ 6	□ 7
29. music is a kind of addiction for me - I couldn't live without it.	□ 1	□ 2	□ 3	□ 4	□ 5	□ 6	□ 7
30. I can identify what is special about a particular piece of music.	□ 1	□ 2	□ 3	□ 4	□ 5	□ 6	□ 7
31. when I have heard a song two or three times, I can usually sing it by myself.	□ 1	□ 2	□ 3	□ 4	□ 5	□ 6	□ 7
32. i often read or search the internet for things related to music.	□ 1	□ 2	□ 3	□ 4	□ 5	□ 6	□ 7
33. i am able to hit the right notes when i sing along to a recording.	□ 1	□ 2	□ 3	□ 4	□ 5	□ 6	□ 7
34. I engage in a lot of music-related activities in my free time.	□ 1	□ 2	□ 3	□ 4	□ 5	□ 6	□ 7
35. i can sing and/or play music from memory.	□ 1	□ 2	□ 3	□ 4	□ 5	□ 6	□ 7
36. I would not describe myself as a musician.	□ 1	□ 2	□ 3	□ 4	□ 5	□ 6	□ 7
37. I can compare two interpretations or versions of the same piece of music and discuss differences.	□ 1	□ 2	□ 3	□ 4	□ 5	□ 6	□ 7
38. I don't like to sing in public because I'm afraid of hitting wrong notes.	□ 1	□ 2	□ 3	□ 4	□ 5	□ 6	□ 7
39. When I sing, I have no idea whether I'm singing right or wrong.	□ 1	□ 2	□ 3	□ 4	□ 5	□ 6	□ 7
40. I have never been praised for my musical ability.	□ 1	□ 2	□ 3	□ 4	□ 5	□ 6	□ 7
41. When someone else sings a song I know, I can sing a second part to it.	□ 1	□ 2	□ 3	□ 4	□ 5	□ 6	□ 7
42. I like to write about music, for example, in Internet blogs or forums.	□ 1	□ 2	□ 3	□ 4	□ 5	□ 6	□ 7

43. i can play ___ instrument/s. (*Whole number*)

44. I have practiced regularly and daily at least one instrument (including singing) for ___ year(s). (*<1 possible*)

45. At the peak of my interest, I practiced my main instrument ___ hour(s) per day. (*average*, *<1 possible*)

46. How would you rate the quality of the headphones you used in this experiment?

□	□	□	□	□
Very poor	Rather poor	average	Rather high	Very high
