# Supplementary material for: Emotionally congruent music and text increase immersion and appraisal
Source: PLoS One. 2023 Jan 12;18(1):e0280019. doi: 10.1371/journal.pone.0280019 (PMC9836297; doi:10.1371/journal.pone.0280019)

Johannes Gutenberg-Universität Mainz • Psychologisches Institut • Wallstraße 3 • 55122 Mainz

PRE-SURVEY **VP-No.___**

V1. How do you feel right now? Please mark the corresponding manikin or a space in between.

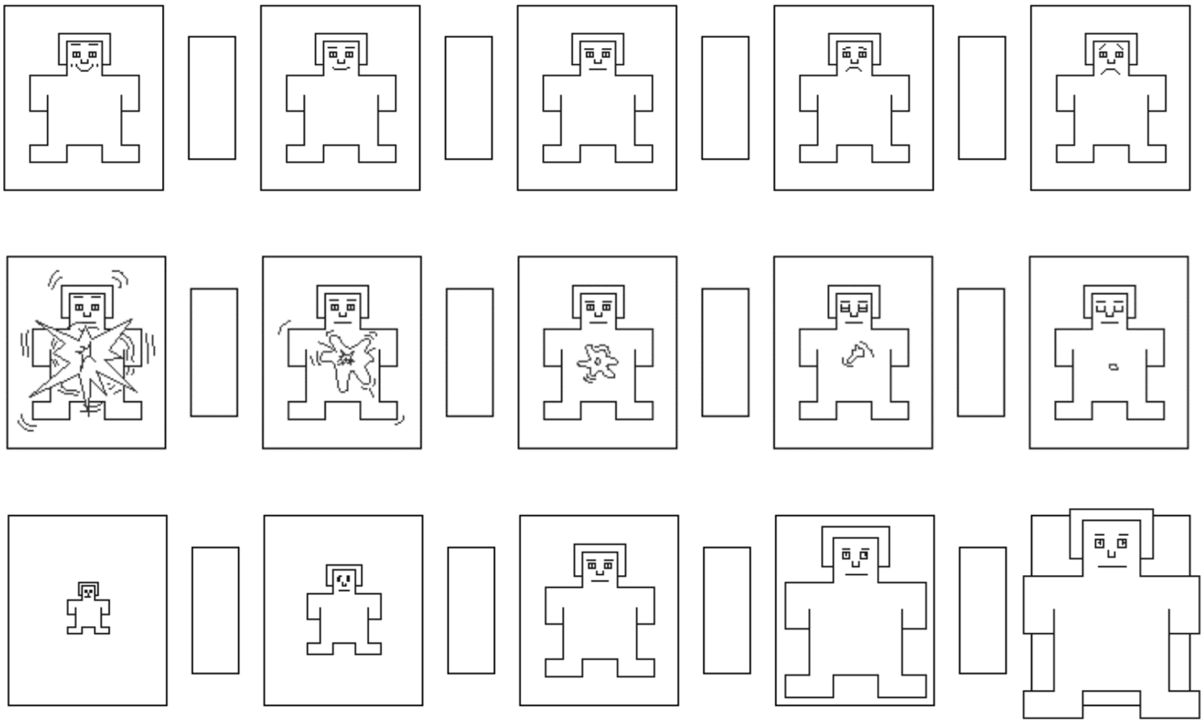

Supplement: S4 Questionnaire — To assess the prevailing mood before the confrontation with any stimuli as a baseline. (PDF) [file pone.0280019.s012.pdf]
