# Supplementary figures and images for: Emotionally congruent music and text increase immersion and appraisal
Source: PLoS One. 2023 Jan 12;18(1):e0280019. doi: 10.1371/journal.pone.0280019 (PMC9836297; doi:10.1371/journal.pone.0280019)

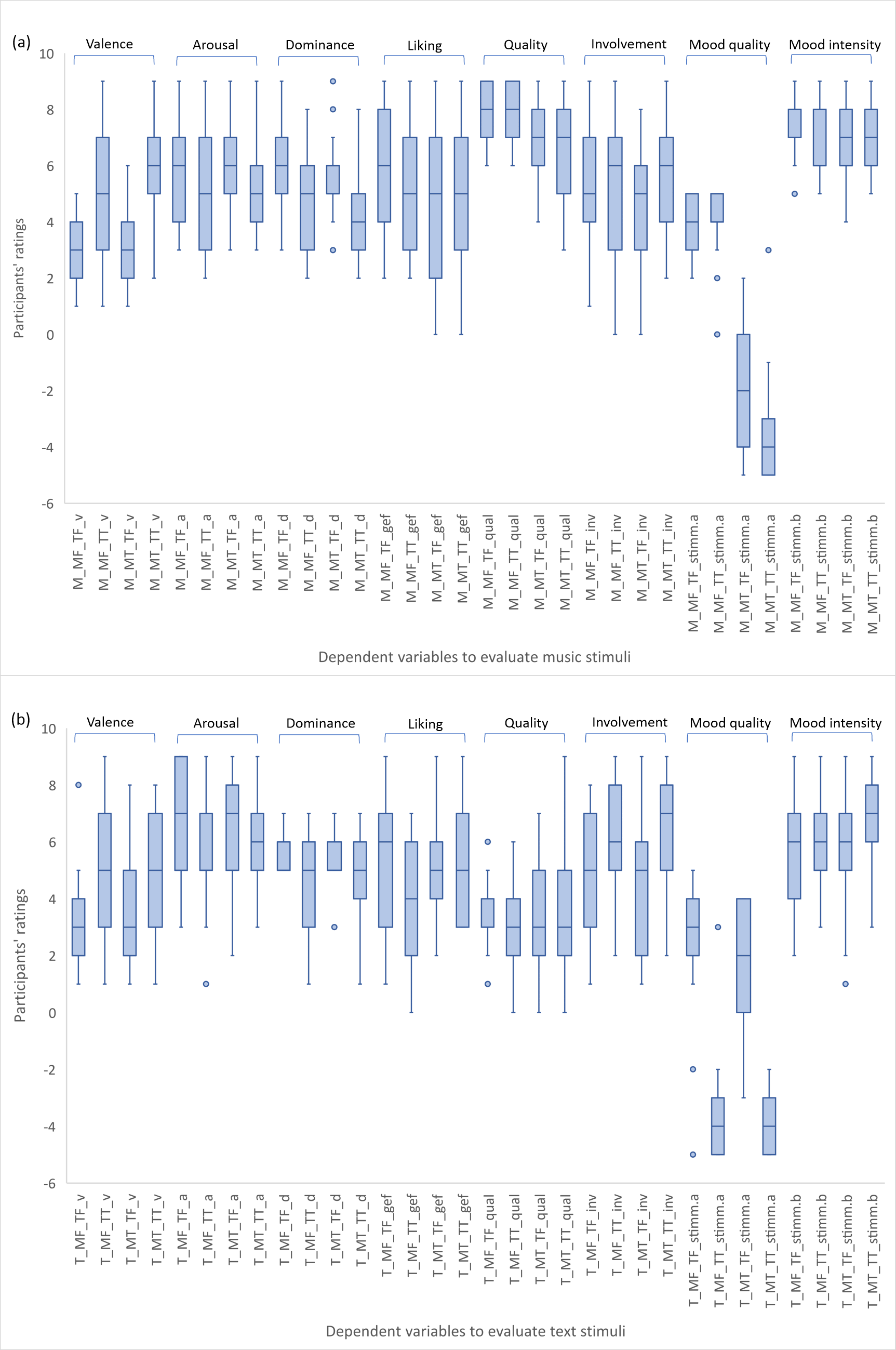

Supplement: S1 Fig — Boxplots to illustrate participants’ ratings of dependent variables concerning a) music and b) text stimuli. (TIF) [file pone.0280019.s014.tif]
