# Supplementary material for: Emotionally congruent music and text increase immersion and appraisal
Source: PLoS One. 2023 Jan 12;18(1):e0280019. doi: 10.1371/journal.pone.0280019 (PMC9836297; doi:10.1371/journal.pone.0280019)
Supplement: Attachment — Submitted filename: MRResponse to reviewers_221216.docx [file pone.0280019.s018.docx]

**PONE-D-22-13721R1 – Response to reviews**

*Dear Dr. Hauck,

Thank you for submitting your very good work!*

*You will notice that reviewers’ comments are good and you satisfied most of their concerns.*

*Based on my own reading, I ask you to give more details on*

***E.1*** *eligibility, stats and a smooth conclusion with further directions for researchers and readers.*

- We have worked on the conclusion and put an emphasis on future directions.

***E.2*** *Also, maybe the authors want to make some parts available on OSF or so,*

- We uploaded the lists of stimuli, the questionnaires, and all data here: DOI 10.17605/OSF.IO/SF47R.
- The specific texts and music files used as stimuli could not be uploaded due to copyright regulations.

*then others will easily replicate your study. It’s basically a few and quick amendments to be made, but nothing worrying. I will be happy to receive your edits and proceed with your study. From now, I am anticipating my sincere “good luck” with this very good one and relevant work. Hope the authors can consider PLOS again.*

*Please submit your revised manuscript by* ***Dec 21 2022 11:59PM.*** *If you will need more time than this to complete your revisions, please reply to this message or contact the journal office at* [*plosone@plos.org*](mailto:plosone@plos.org)*. When you're ready to submit your revision, log on to* [*https://www.editorialmanager.com/pone/*](https://www.editorialmanager.com/pone/) *and select the 'Submissions Needing Revision' folder to locate your manuscript file.

Please include the following items when submitting your revised manuscript:*

- *A rebuttal letter that responds to each point raised by the academic editor and reviewer(s). You should upload this letter as a separate file labeled 'Response to Reviewers'.*
- *A marked-up copy of your manuscript that highlights changes made to the original version. You should upload this as a separate file labeled 'Revised Manuscript with Track Changes'.*
- *An unmarked version of your revised paper without tracked changes. You should upload this as a separate file labeled 'Manuscript'.*

***E.3*** *If you would like to make changes to your financial disclosure, please include your updated statement in your cover letter.*

- We’ve done it accordingly, see Cover Letter

*Guidelines for resubmitting your figure files are available below the reviewer comments at the end of this letter. If applicable, we recommend that you deposit your laboratory protocols in* [*protocols.io*](http://protocols.io) *to enhance the reproducibility of your results.* [*Protocols.io*](http://Protocols.io) *assigns your protocol its own identifier (DOI) so that it can be cited independently in the future. For instructions see:* [*https://journals.plos.org/plosone/s/submission-guidelines#loc-laboratory-protocols*](https://journals.plos.org/plosone/s/submission-guidelines#loc-laboratory-protocols)*. Additionally, PLOS ONE offers an option for publishing peer-reviewed Lab Protocol articles, which describe protocols hosted on* [*protocols.io*](http://protocols.io)*. Read more information on sharing protocols at* [*https://plos.org/protocols?utm_medium=editorial-email&utm_source=authorletters&utm_campaign=protocols*](https://plos.org/protocols?utm_medium=editorial-email&utm_source=authorletters&utm_campaign=protocols)*.

We look forward to receiving your revised manuscript.*

Kind regards,

Thiago P. Fernandes, PhD
Academic Editor
PLOS ONE

**Journal Requirements:**

***E.4*** *Please review your reference list to ensure that it is complete and correct. If you have cited papers that have been retracted, please include the rationale for doing so in the manuscript text, or remove these references and replace them with relevant current references. Any changes to the reference list should be mentioned in the rebuttal letter that accompanies your revised manuscript. If you need to cite a retracted article, indicate the article’s retracted status in the References list and also include a citation and full reference for the retraction notice.*

- We have reviewed the reference list again, but we did not find any papers that have been retracted. Is there a particular paper you have in mind? Maybe the issue is with the dates on e-publication ahead of the print issue? We have updated to print issue citation where available. Further, of the references in ‘submitted’ status had been published in the meantime. We also made some changes to journal titles, now using short titles.
- We also corrected the numbering of tables.

**Reviewer #1: Manuscript ID: PONE-D-22-13721_R1 2nd Round**

The authors did an excellent job clarifying all the questions I have raised in my previous round of review. Currently, this paper entitled ‘Emotionally congruent music and text increase immersion and appraisal’, is a well-written, timely piece of research that examined how emotions in texts are related to emotions in the music listened to while reading.

Overall, this is a timely and needed work. It is well researched and nicely written, and describes in detail distinct patterns of perceived music-text-fit, demonstrating how the more similar music and text were evaluated in mood, the better the stimuli were regarded to match.
I believe that this paper does not need a further revision, therefore the manuscript meets the Journal’s high standards for publication.
I am always available for other reviews of such interesting and important articles.
Thank You for your work.

**Reviewer #3: Thank you for your very diligent and careful work. I only have a few**

**suggestions, but nothing time consuming:**

***R3.1*** *Regarding the Methods, more details are need. For example, the authors need to explain the timeline, screening, confounding variables, the Cronbach values for any scale, all details of the analyses.*

- We added one sentence about the timeframe in which data were gathered. (l. 374)
- All details of the analyses can be found in the Supplementary Material.

***R3.2*** *More specifically, details on eligibility is needed: were the patients free of cognitive disorders? (10.3389/fpsyt.2013.00182),*

- We cannot be 100 % certain. We added a sentence about the non-performance of cognitive tests (l. 248-250)

***R3.3*** *had normal visual acuity (10.1016/j.jpsychires.2022.03.014 and 10.1016/j.clinph.2005.06.013),*

- We relied on self-reports as to visual and hearing acuity. We added the word “self-reported” to the respective sentence (see l. 244)

***R3.4*** *had no previous contact with substances (10.3389/fpsyg.2018.00288),*

- We added a sentence about the non-testing of substance use. (l. 248-250) In our understanding, it is not highly relevant for this kind of experiment on multisensory perception, as it does not include any clinical trials.

***R3.5*** *if they had previous contact with any other perceptual task before or were naive to them;*

- We added two sentences about this, see l. 240-243

***R3.6*** *> Use more illustrative graphs to demonstrate your findings;*

- After having thoroughly evaluated different kinds of graphics to further illustrate our findings, we concluded that the existing three graphs in conjunction with tables are the most comprehensible and simple way to show our findings. Nothing would be gained but some information lost if we were to replace the tables by graphics.

***R3.7*** *> Use effect sizes and CIs for the analysis; Use the corrected p-value;*

- Effect sizes (η²) are included in the results tables (see Tables 1, 2, S4 – S8). *p*-values as results of post-hoc analyses were LSD-corrected (we added this information to the text in l. 395). Other corrections were not indicated.

***R3.8*** *> Boxplots with individuals values are more representative of the data and the sample (10.1038/nmeth.2813)*

- Yes, agreed. As this would bloat the manuscript, we added boxplots to the supplementary materials (see S1 Figure) and refer to them at the beginning of the results section (see l. 386-378).
